# Impact of Reduced School Exposure on Adolescent Health Behaviors and Food Security: Evidence From 4‐Day School Weeks

**DOI:** 10.1111/josh.13095

**Published:** 2021-10-06

**Authors:** Emily J. Tomayko, Paul N. Thompson, Madeleine C. Smith, Katherine B. Gunter, John M. Schuna

**Affiliations:** ^1^ Assistant Research Professor, (emilytomayko@montana.edu), Center for American Indian and Rural Health Equity, Montana State University, 2155 Analysis Drive Bozeman MT 59718. USA; ^2^ Associate Professor, Economics, (paul.thompson@oregonstate.edu), School of Public Policy, Oregon State University, 340 Bexell Hall Corvallis OR 97331. USA; ^3^ Predoctoral Fellow, (madeleine.smith@econ.uzh.ch), Department of Economics, University of Zurich, Schönberggasse 1 CH‐8001 Zürich Switzerland.; ^4^ Professor and Extension Specialist, Kinesiology, (kathy.gunter@oregonstate.edu), College of Public Health and Human Sciences, Oregon State University, 2631 SW Campus Way Corvallis OR 97331. USA; ^5^ Associate Professor, Kinesiology, (john.schuna@oregonstate.edu), College of Public Health and Human Sciences, Oregon State University, 2631 SW Campus Way Corvallis OR 97331. USA

**Keywords:** adolescent health, health behaviors, schools, diet, pediatric obesity, physical education, food insecurity, risk‐taking, marijuana use

## Abstract

**BACKGROUND:**

Four‐day school week (FDSW) use has increased substantially among US districts in recent years, but limited data exist on health impacts of this school schedule. This study examined associations of reduced school exposure via FDSWs with adolescent health and risk behaviors, obesity, and food security.

**METHODS:**

Self‐report data from 8th‐ and 11th‐grade students from the Oregon Healthy Teens survey across 5 survey years (odd years 2007‐2015, total N = 91,860‐104,108 respondents depending on the survey question) were linked to a FDSW indicator. Regression analyses controlling for student and school characteristics compared outcomes between students in 4‐ and 5‐day schools overall (without school fixed effects) and outcomes associated with switching to a FDSW (with school fixed effects).

**RESULTS:**

When controlling for multiple student‐ and school‐level factors, we observed adolescents in FDSW schools report they consume sugar sweetened beverages more frequently and water less frequently, have access to fewer days of physical education, are more likely to be food insecure, and are more likely to report the use of any drugs and specifically marijuana than 5‐day school week students.

**CONCLUSIONS:**

Limiting exposure to the school environment via FDSWs may impact adolescent health behaviors, including diet, physical activity, and drug use.

The school environment can profoundly influence children into adolescence and adulthood.^1^, ^2^, ^3^ School settings enable increases in student knowledge; nurture students' socioemotional development by providing a structured social environment for interactions with peers, teachers, and administrators; and promote healthy behaviors via access to school meal programs and physical activity opportunities. Previous work suggests that skill‐building and healthy behaviors fostered through exposure to this structured school environment may have lasting impacts on students as they make college‐going and labor market decisions.^4^

Despite identified benefits of the school environment, the last 2 decades have seen unprecedented declines in state education funding. These declines have forced many US school districts to consider cost‐cutting measures that may alter the quality and/or quantity of students' exposure to the school environment. School districts have increasingly adopted four‐day school weeks (FDSWs) as a cost‐cutting measure that eliminates 1 day per week while lengthening the 4 remaining school days. This schedule is employed predominately in rural districts, with the majority of districts citing financial motivations for making the switch.^5^ These districts have lower enrollments and higher per‐student expenditures, which, along with transportation and other logistical issues, may partially explain the higher use of FDSW schedules in rural areas.^5^ Despite the lengthening of the 4 remaining school days concomitant with FDSW use, overall time in school is significantly lower, decreasing access to critical learning, socioemotional, and health behavior supports.^5^ Four‐day school weeks have been shown to produce cost savings^5^ and achievement impacts^6^, ^7^ that are comparable to other cost‐savings approaches,^8^ such as increasing class size and implementing year‐round schedules. However, students' reduced school exposure via FDSWs may have repercussions on other child outcomes that are not present for other cost‐saving measures.

The potential repercussions associated with FDSW use could span multiple domains. For example, data show that earlier school start times, a strategy often employed to increase school day length concurrent with FDSW implementation, has a detrimental effect on adolescent sleep and behavioral health.^9^ The loss of food services once per week may impact child dietary behaviors and is particularly relevant given 57% of children in FDSW schools were free‐ and reduced‐lunch eligible in 2018‐2019.^5^ Children accumulate at least 70% of their daily moderate‐to‐vigorous physical activity during the school day, the majority of which comes during recess and physical education.^10^, ^11^, ^12^, ^13^ For adolescents who may not participate in recess, physical education exposure may contribute significantly to overall physical activity levels and related health outcomes. These changes to diet and physical activity via FDSWs may consequently impact obesity risk.

The lack of structure and supervision on the non‐school weekday may affect other behaviors in adolescents, such as substance use, sexual behavior, and crime. A recent study^14^ showed both positive (decreased substance use and screen time, increased physical activity) and negative (increased sexual activity, decreased sleep and breakfast consumption) associations with FDSW exposure among teens in Colorado, while a separate Colorado‐based study showed increases in juvenile crime associated with FDSW use.^15^ At a time of economic distress when education allocations may be threatened, it is critical to gain more insights into the effects of FDSWs on students' health and wellbeing to inform decision makers.

The objective of the current study was to assess adolescent health and risk behaviors, obesity, and food security among 8th‐ and 11th‐grade students in 4‐ versus 5‐day schools in Oregon, which ranks fourth in the United States for states employing FDSWs. In 2018‐2019, 80 Oregon districts employed FDSWs in at least 1 school, representing 40.6% of the state's total districts. In Oregon FDSW schools, total time is school is decreased by 3‐4 hours per week compared to Oregon 5‐day schools.^6^ We hypothesized that detrimental trends in health and risk variables, such as less physical activity and more substance use, would be observed in association with FDSW use given the reduced exposure to the school environment.

## METHODS

### Dataset: Longitudinal 4‐Day School Week Database

We previously compiled a comprehensive dataset of FDSW use in Oregon via reviews of the Oregon Department of Education, Oregon School Board Association, and Oregon Health Authority websites. A systematic phone/email survey verified which school districts in Oregon had a FDSW and when the school schedule was implemented. From this verification process, we created a dummy variable indicating whether the school used a FDSW in a given year between 2007 and 2015.

### Participants

We combined this FDSW database with data from the Oregon Healthy Teens (OHT) survey, which is conducted by the Oregon Health Authority in collaboration with the Oregon Department of Education. The OHT survey is a validated and reliable instrument^16^, ^17^ that incorporates 2 surveys that were used prior to 1999, the Youth Risk Behavior Survey and the Student Drug Use Survey. The OHT survey is administered once every 2 years to collect self‐reported information on demographics and health behaviors of students in 8th and 11th grade in Oregon. Participation is voluntary and anonymous. The sampling frame for the survey utilized Oregon Department of Education's Fall Membership Report for a given school year and includes Oregon public schools that have 8th‐ and/or 11th‐grade enrollment. The sampling frame is intended to produce valid state and county‐level estimates. After eligible schools within the sampling frame are stratified by county, schools are sampled in proportion to their size within a given county. If a selected school declines participation, a comparable school is selected based on enrollment and other factors. Resulting state‐level data are then weighted according to enrollment numbers.

We obtained data from the 2007, 2009, 2011, 2013, and 2015 OHT surveys. The numbers of participating schools and student respondents per year are listed in Table 1. Across the 5 survey years included in this study, the number of respondents per question ranged from 91,860 to 104,108 students. In total, 362 unique schools, including 39 four‐day and 323 five‐day schools, participated over the 5 years.

**Table 1:** Number of Participating Schools and Student Respondents per Oregon Healthy Teens Survey Year in 4‐ and 5‐Day School Schedules

Survey Year	Four‐Day Schools	Five‐Day Schools	Total Schools	Four‐Day Students	Five‐Day Students	Total Students
2007	28	210	238	1254	23,541	24,795
2009	12	100	112	421	13,046	13,467
2011	10	79	89	377	10,446	10,823
2013	24	191	215	1276	25,638	26,914
2015	26	196	222	1372	26,737	28,109

### Instrumentation

From the survey, we used variables on nutrition, physical activity, obesity, food security, substance use, and other risk behaviors. Specifically, we included the number of times per day consuming fruit, vegetables, sugar sweetened beverages, soda, milk, and water during the last 7 days; number of times eating breakfast during the last 7 days; number of physical education days/week; frequency of days physically active for at least 60 minutes during the last 7 days; screen time use on an average school day; self‐report height and weight; food security during the past 12 months; use of any drugs, alcohol, marijuana, cigarettes, and prescription drugs without a prescription during the past 30 days; driving after drinking alcohol during the past 30 days; engaging in sexual intercourse during the last 30 days; ever been a victim of forced sex; and engaging in a physical fight over the last 12 months. The nutritional and physical activity behavior variables were created as continuous variables that provide a measure of the number of times per day of engagement in the particular behavior. If a range of frequency was indicated, responses were converted to fractional times per day measures using the median values of these ranges; for example, 1‐3 times/day was converted to 2 times/day. The food security, overweight/obesity, substance use, sexual health, and violence variables were created as dichotomous variables that indicate any versus no engagement in the particular behavior. We also used demographic variables, such as student race/ethnicity and the region where the school is located. Differences in survey design across years were resolved by dropping unrepeated questions and unifying variable names. We generated a variable to account for survey year and compiled the datasets from different years into 1 dataset.

### Procedure

The OHT survey was administered by participating schools in the spring of odd‐numbered years. The survey was administered as either paper‐and‐pencil or using a web‐based version and was completed during a single class period. At the individual level, both parents and students were provided the opportunity to opt out of any given survey using an active notification/passive consent model. The current study consisting of secondary analysis of the OHT survey data linked to our FDSW dataset was approved by the Oregon State University Institutional Review Board (Study #8919).

### Data Analysis

We used regression analysis to account for potential differences due to student characteristics or regional and time variation in the outcomes. Specifically, we estimated the following repeated cross‐sectional regression specification using students from all school districts included in the dataset across the 5 survey years:

(1) $$Y_{\mathrm{ist}}=\alpha +\beta_{1}{\text{FDSW}}_{\mathrm{st}}+\gamma X_{\mathrm{ist}}+\theta_{s}+\lambda_{t}+\epsilon_{\mathrm{ist}}$$

where $Y_{\mathrm{ist}}$ is a survey response to questions relating to each health/risk behavior. For the dichotomous dependent variables, this specification amounts to a linear probability model. The ${\text{FDSW}}_{\mathrm{st}}$ variable is a dummy variable equal to 1 if the school was operating on a FDSW schedule during school year t. The $X_{\mathrm{ist}}$ vector contains student characteristics, including age, race, and sex, $\theta_{s}$ is a school fixed effect, $\lambda_{t}$ is a school year fixed effect, and $\epsilon_{\mathrm{dt}}$ is an idiosyncratic error term clustered at the school level.

We conducted this analysis in 2 ways. First, we ran the regression model without school fixed effects, which is similar to the methodological approach taken by Israel et al.^14^ This across‐school analysis gives the mean differences across the 4‐ and 5‐day school week schedules when controlling for student, time, and regional variation in these health behaviors. As students are nested in schools, however, the preferred estimates come from estimating Equation (1) with school fixed effects included. This approach allows for the within‐school change in these behaviors to be measured after the school adopts a FDSW, in other words, the effect of switching to a FDSW. Moreover, this approach eliminates issues of heterogeneity in the underlying prevalence of these behaviors across schools that would bias estimates of the FDSW effects. Although this analysis represents a more robust approach, the OHT data set only contains 8 schools that switched to a FDSW during our study period. Thus, although all observations are included in this regression model, the FDSW effects resulting from this analysis are identified from this smaller subset of FDSW switchers.

## RESULTS

### Demographics

Table 2 presents descriptive statistics for the students included across all survey years. Mean age across all participating students was 15.01 ± 1.6 years; the average age was slightly but significantly higher in 4‐ compared to 5‐day schools. Percent of female students was approximately half across all participants. Overall, students included in the sample are predominately white at approximately 86% of the total sample. However, significantly more non‐white students attended 5‐day schools. Sexual orientation was significantly different between school schedules, with a higher percentage of students in FDSWs identifying as heterosexual. There was no significant difference in free and reduced lunch eligibility between school schedules.

**Table 2:** Demographics of Student Respondents in 4‐ Versus 5‐Day School Schedules (2007‐2015)

	Four‐Day (N = 4700)	Five‐Day (N = 99,408)	p‐Value
Age, years (mean ± SD)	15.3 ± 1.6	15.0 ± 1.6	<.001
Grade (%)			
8th	48.3 ± 50.0	56.0 ± 49.6	<.001
11th	51.7 ± 50.0	44.0 ± 49.6	<.001
Sex (% female)	49.0 ± 50.0	50.5 ± 50.0	.049
Race/ethnicity (%)			
Non‐Hispanic white	78.9 ± 40.8	71.2 ± 45.3	<.001
Non‐Hispanic black	0.8 ± 9.0	2.4 ± 15.3	<.001
Hispanic	5.5 ± 22.8	10.9 ± 31.1	<.001
Other/multi‐racial	19.0 ± 39.2	23.3 ± 42.3	<.001
Sexual orientation (%)			
Heterosexual	92.5 ± 26.4	90.7 ± 29.0	<.001
LGB	7.5 ± 26.4	9.3 ± 29.0	<.001
Free/reduced lunch eligibility (%)	40.0 ± 49.0	38.5 ± 48.7	.155

### Health Behaviors and Health‐Related Outcomes

Table 3 presents raw descriptive statistics for the various outcome variables used in the study. Table 4 displays regression results from Equation (1) both excluding and including school fixed effects. The model that excludes school fixed effects allows for an across‐school comparison of all observations of students in 4‐ versus 5‐day schools, controlling for student‐level factors and region. Given that 5 survey years were included in this analysis, the model that includes school fixed effects allows for dynamic within‐school comparison related to the switch to a FDSW while controlling for the same student‐level factors and region. The FDSW may be correlated with many of these student and school‐level factors; therefore, the regression results that include school fixed effects are likely more informative in regard to the overall impacts of the FDSW. The findings presented in Table 4 are described in the following sections.

**Table 3:** Raw Data Comparing Student Health and Behavior Responses in 4‐ Versus 5‐Day Schedules

	Four‐Day	Five‐Day	p‐Value
Nutritional behaviors, past 7 days (mean ± SD)			
Number of times consuming …			
Fruits/day	0.93 ± 0.96	1.05 ± 1.03	<.001
Vegetables/day	1.48 ± 1.47	1.53 ± 1.55	.029
Sugar sweetened beverages/day	1.93 ± 2.06	1.59 ± 1.98	<.001
Soda/day	0.62 ± 0.85	0.52 ± 0.78	<.001
Glasses of milk/day	1.11 ± 1.25	0.90 ± 1.15	<.001
Water/day	1.70 ± 1.73	1.66 ± 1.71	.086
Days eating breakfast	5.95 ± 0.69	5.98 ± 0.70	.003
Food insecure (% yes, past 12 months)	17.6 ± 38.1	17.2 ± 37.8	.563
Physical activity behaviors (mean ± SD)			
Physical education days (avg school week)	3.15 ± 1.97	3.59 ± 2.27	<.001
Days physically active ≥60 minutes (past 7 days)	4.71 ± 2.22	4.44 ± 2.30	<.001
Screen time hours/day (avg school day)	3.23 ± 2.37	3.52 ± 2.37	<.001
Overweight/obese (%), self‐report	28.2 ± 45.0	25.0 ± 43.3	<.001
Substance use (% yes, past 30 days) (mean ± SD)			
Any drug use	19.5 ± 39.7	20.1 ± 40.1	.428
Any marijuana use	13.6 ± 34.3	14.8 ± 35.5	.032
Any alcohol use	29.3 ± 45.5	25.6 ± 43.7	<.001
Any cigarette use	11.9 ± 32.4	8.5 ± 27.9	<.001
Took prescription drug without a prescription	6.2 ± 24.2	5.6 ± 22.9	.046
Drove after drinking alcohol	5.0 ± 21.7	4.5 ± 20.7	.221
Sexual health			
Had sexual intercourse (past 3 months)	21.8 ± 41.2	16.8 ± 37.4	<.001
Ever been victim of forced sex	6.2 ± 24.1	5.7 ± 23.3	.370
Violence			
Been in a physical fight (past 12 months)	11.9 ± 32.3	11.7 ± 32.1	.735

Depending on the question, number of observations ranged from 2563‐4700 for 4‐day school respondents and 50,214‐99,408 for 5‐day school respondents.

**Table 4:** Regression Analysis Comparing Student Health and Behavior Responses in 4‐ Versus 5‐Day Schedules

	Without School Fixed Effects		With School Fixed Effects	
	Coefficient ± SE	p‐Value	Coefficient ± SE	p‐Value
Nutritional behaviors (past 7 days)				
Frequency of fruits/day	−0.045 ± 0.025	.075	−0.057 ± 0.066	.386
Frequency of vegetables/day	0.006 ± 0.036	.875	−0.042 ± 0.091	.643
Frequency of sugar sweetened beverages/day	0.235 ± 0.113	.039	0.092 ± 0.045	.041
Frequency of soda/day	0.063 ± 0.029	.028	−0.014 ± 0.062	.826
Frequency of milk/day	0.086 ± 0.026	.001	−0.025 ± 0.041	.542
Frequency of water/day	−0.039 ± 0.032	.227	−0.139 ± 0.052	.008
Days eating breakfast	−0.019 ± 0.019	.333	0.002 ± 0.057	.970
Food insecure (% yes, past 12 months)	−0.011 ± 0.012	.366	0.085 ± 0.006	<.001
Physical activity behaviors				
Physical education days (avg school week)	−0.391 ± 0.145	.008	−0.882 ± 0.202	<.001
Days physically active ≥60 minutes (past 7 days)	0.115 ± 0.065	.075	−0.228 ± 0.255	.372
Screen time hours/day (avg school day)	−0.187 ± 0.061	.002	−0.185 ± 0.203	.364
Overweight/obese (%), self‐report	0.030 ± 0.010	.002	0.012 ± 0.026	.663
Substance use (past 30 days)				
Any drug use	−0.013 ± 0.013	.316	0.107 ± 0.036	.003
Any marijuana use	−0.019 ± 0.010	.048	0.065 ± 0.025	.010
Any alcohol use	−0.002 ± 0.013	.873	0.028 ± 0.039	.475
Any cigarette use	0.018 ± 0.010	.059	−0.004 ± 0.023	.873
Took prescription drug without a prescription	0.000 ± 0.005	.959	0.017 ± 0.011	.123
Drove after drinking alcohol	−0.010 ± 0.005	.071	−0.010 ± 0.017	.575
Sexual health				
Had sexual intercourse (past 3 months)	0.020 ± 0.013	.130	−0.052 ± 0.046	.265
Ever been victim of forced sex	0.006 ± 0.006	.253	0.017 ± 0.014	.239
Violence				
Been in a physical fight (past 12 months)	0.004 ± 0.007	.557	−0.001 ± 0.022	.965

#### Diet, physical activity, and related factors

There were no significant differences in the frequency of fruit, vegetable, water, or breakfast consumption between students in 4‐ and 5‐day schools. Students in 4‐day school week schools reported consuming more sugar sweetened beverages (p = .039) and soda (p = .028). When school fixed effects were considered to examine the effect of switching to a FDSW, the effect of significantly higher sugar sweetened beverage intake among FDSW students remained. While soda intake, specifically, was no longer greater after switching to a FDSW, there was a slight but significant difference in frequency of water consumption, with students who had switched to a FDSW reporting drinking water less frequently (p = .008).

Days of physical education per week were significantly different between the 2 school schedules, with an average of 3.6 days per week reported by students in 5‐day school week schools and 3.2 days per week reported by students in FDSW schools (p = .008). Although not significant, there was a trend toward a greater number of days students reported being physically active for at least 60 minutes in 4‐ compared to 5‐day schools (p = .075). Hours of reported screen time on the average school day were significantly less among students in FDSW schools compared to 5‐day schools (p = .002). With school fixed effects included, the days of physical education remained significantly lower for 4‐day students (p < .001), while there were no differences in days physically active or screen time use associated with switching to a FDSW. Students were more likely to report a height and weight that would be classified as overweight or obese in FDSW schools (p = .002); there was no longer a difference in obesity prevalence when school fixed effects were included. However, there was a significant increase in food insecurity associated with the switch to a FDSW (p < .001).

#### Risk behaviors

Regarding substance use over the past 30 days, reported marijuana use was significantly lower among FDSW students (p = .048) with a trend for higher cigarette use (p = .059). With the inclusion of school fixed effects, the switch to a FDSW was associated with significantly greater use of any drug (p = .003) and significantly higher use of marijuana among students at schools that switched to a FDSW (p = .010). There was no difference in alcohol or prescription drug use without a prescription between school models with and without school fixed effects included. Approximately 22% of students in FDSW schools reported having sexual intercourse within the last 30 days compared to 17% in 5‐day schools; however, this difference was not significant in either regression model. There also was no difference in the likelihood of having been a victim of forced sex or reported instances of being involved in a physical fight over the past year between school schedules in either regression model.

## DISCUSSION

Our findings of both positive and negative effects associated with FDSW use contributes to an emerging body of evidence about the health impacts of reduced school exposure via FDSWs. Only recently have any health effects of this school schedule been described, with a study comparing adolescents in 4‐ and 5‐day Colorado schools also finding both positive and negative impacts associated with FDSWs.^14^ The study described here has a very similar premise to the Colorado study but analyzes these FDSW impacts in a different state policy setting, allowing for comparison of our results to those found for Colorado. In our study, we used 2 models: one not accounting for school fixed effects that considered all 4‐ versus 5‐day respondents and one that accounted for school fixed effects to look at the impacts of switching to a FDSW, made possible by the inclusion of multiple survey years. When using similar methods to Israel et al by not accounting for school fixed effects,^14^ our results are quite comparable to those found in Colorado. These include students in 4‐day schools reporting they are less likely to use marijuana (p = .048); less likely to drive impaired by alcohol (trend, p = .071); have fewer hours of daily non‐school screen time (p = .002); and have more days of physical activity (trend, p = .075). Our results suggest the prevalence of these behaviors may be quite similar across the primarily rural, FDSW adopters in both Colorado and Oregon.

Due to differences in prevalence of these health behaviors across school districts, however, we stress caution in attributing the cross‐sectional differences noted in the non‐school fixed‐effect models—both in Oregon and Colorado—directly to the impact of FDSWs. Longitudinal or repeated cross‐sectional data that allow for within‐school estimates to be generated, like in the current study, are essential for accounting for this heterogeneity in prevalence across schools. As the school fixed‐effect model results suggest, accounting for these differences in underlying prevalence rates yields quite different estimates regarding the effects of switching to the FDSW on these behaviors. When controlling for multiple student‐ and school‐level factors, we observed that 8th‐ and 11th‐grade students in schools that switched to a FDSW report that they consume sugar sweetened beverages more frequently and water less frequently, have access to fewer days of physical education, are more likely to be food insecure, and are more likely to report the use of any drugs, and specifically marijuana, than 5‐day school week students. Thus, applying these methods to similar data in Colorado and other FDSW states will be essential for generalizing the effects of switching to the FDSW across different state contexts.

Studies have shown declines in healthy behaviors like sleep and physical activity during non‐school versus school time.^18^, ^19^, ^20^ These data suggest that repeated exposure to 3 consecutive non‐school days may have similar effects. Older children, such as the 8th and 11th graders surveyed here, also have increasing autonomy over food choice^21^ and physical activity participation^22^ compared to younger children. In this study, we showed significantly more frequent sugar sweetened beverage consumption and less frequent water consumption among 4‐ versus 5‐day week students, although other differences in food and beverage consumption were not observed. These results suggest adolescents may be making these unhealthier drink choices when not exposed to the supervision of the structured home or school environments. These findings also may be linked to the lack of access to potentially healthier drinking options at school 1 additional day per week. However, it remains unclear if and how these changes are related to health outcomes, such as obesity risk, and if there are other subtle changes to dietary intake that are not captured by the types of questions used in the OHT survey, as food frequency questions may not be the most accurate reflection of actual intake.^23^ Although there was a significant difference in obesity for students in 4‐ versus 5‐day schools observed in the overall sample, this effect was not maintained when examining the within school effects. This suggests that the results are primarily driven by differences in obesity prevalence across 4‐day and 5‐day schools, particularly in rural areas where obesity rates are known to be higher.^24^

The finding of significantly fewer days of physical education reported by students in FDSW schools is not surprising given they are in school 1 fewer day per week than their counterparts in 5‐day schools. Moreover, high school students may not be required to participate in physical education every year, suggesting adolescents in FDSW schools may be at higher risk for inadequate school day physical activity. We previously showed that among elementary students, minutes of physical education were actually increased in FDSW schools both in Oregon^25^ and nationally.^5^ Of note, there are different physical education requirements at primary versus secondary levels in Oregon that confound comparisons between physical education exposure among adolescents and elementary students in 4‐ versus 5‐day schools. More data are needed to clarify these associations. Ample research shows that school‐day physical activity, and particularly physical education, contribute significantly to child physical activity; consequently, the inadequate levels of physical activity at school are likely not being compensated for in the home environment. Thus, promoting physical activity both at school and in the home remains critical for health promotion efforts among adolescents.

The finding of increased self‐reported food insecurity among children after switching to FDSW schedules in Oregon is of particular concern. FDSW schedules are implemented predominately in rural areas, where rates of poverty and household food insecurity are higher compared to urban areas.^26^ FDSWs may shift the burden of childcare, food provision, and other services from school districts to families and available community supports, which may require wholesale changes to the distribution of household financial resources. For example, the loss of school food services once a week may impact household food allocation, which could potentially explain the increased risk for food insecurity. The non‐school weekday also may present childcare challenges for families, leading to altered family labor force participation^27^ and/or increased childcare costs. The mechanisms related to increased food insecurity risk among children after switching to a FDSW schedule represent an important area for future research to determine how school‐, community‐, and home‐based factors interact to support or hinder food access for rural families. In a previous study of rural elementary‐age children in Oregon, we also found that rural families experiencing food insecurity reported low readiness to provide their children physical activity *outside* of the school setting,^28^ highlighting that school day physical education and other physical activity opportunities are critical for these children, particularly in the context of limited household resources. Although these relationships have not been examined among adolescents, it is likely the increased risk of obesity for rural children may be impacted by a combination of food insecurity and lack of opportunities for physical activity, among other factors. These variables warrant further investigation among children and families exposed to the FDSW schedule.

Our data showed increased reported drug use and specifically marijuana use among students associated with the switch to a FDSW schedule; this finding supports our hypothesis that additional unstructured and potentially unsupervised time created by FDSW schedules may lead to increases in risky behaviors. These findings align with previous research showing a spike in adolescent crime in areas that switched to a FDSW.^15^ Although this finding contrasts with what was shown for FDSW adolescents in Colorado, this is very likely due to the models used in the study as described above. While families with elementary‐age children may choose to remain at home or seek childcare arrangements for the non‐school weekday, families may choose to leave older children home alone. This approach has been associated with detrimental mental health outcomes,^29^, ^30^, ^31^ which also may contribute to increases in unhealthy or risky behaviors in addition to the increased unsupervised time. More data on how families are choosing to supervise their adolescent children on the non‐school weekday could provide insight into these observed increases in risky behavior among students in schools that switched to a FDSW.

### Limitations

This study is limited by the self‐report nature of the OHT survey; however, precautions were taken during data cleaning by the Oregon Health Authority to account for inconsistent response patterns and probable dishonest or exaggerated responses. Also, although the majority of questions were asked across all years included in these analyses, a few were not asked every time. Endogeneity and selection into the FDSW by these schools is problematic in identifying any differences as causal. Although our study was strengthened by the inclusion of multiple survey years, which allowed for an analysis of the impact of switching to a FDSW, these effects were driven primarily by a small number of schools (N = 8). Adding more survey waves of the OHT or similar surveys from other FDSW states will allow for more FDSW switchers to be included in these analyses and allow appropriate causal inference validity checks, such as parallel trends, to be assessed in this context.

### Conclusions

If taken at face value, these results suggest the choice of school schedule may have important implications for adolescent health and risk behaviors. Although multiple outcomes were significantly different between school schedules in the present study, none of the differences were particularly large. It may be possible that small changes across multiple dimensions, like diet, physical activity, and substance use, result in a shift in behavior patterns that contribute to changes in health outcomes. However, the overall impact of these small changes on functional outcomes, such as disease risk, remains unknown. As school districts consider alternate school schedules among continuing economic pressure and other emergent issues that may decrease school exposure (eg, COVID‐19 pandemic, severe weather), these data represent an important step toward considering student health in policy‐making decisions. While districts may be forced to choose among a variety of suboptimal cost‐cutting strategies, the data presented here may help guide policy decisions regarding the structure and implementation of 4‐day school weeks, should that strategy be employed.

## IMPLICATIONS FOR SCHOOL HEALTH

The primary drivers for adoption of FDSWs nationally are cost‐savings; attendance issues related to transportation, athletic events, and/or medical appointments; and other rural‐specific issues, such as teacher retention and family farming/ranching commitments. Given the findings of the current study, we echo the recommendation of Israel et al^14^ that student health also should be considered when either making the decision to switch to a FDSW or in choosing how to structure the school schedule once a FDSW is adopted. It is likely that differences in adoption, structure, and implementation of FDSWs across states will result in differential impacts of FDSWs on adolescent health and health behaviors.

The lack of supervision in a formal school setting created by the FDSW schedule may be compensated for by remedial and/or enrichment programs offered on the non‐school weekday, an approach practiced by some current FDSW districts.^5^ However, given that the majority of school districts that have adopted a FDSW cite financial motivations for making the switch, it is unlikely there will be adequate funding to provide resources to children on the non‐school weekday in many districts. Schools may look to partner with community organizations, such as local libraries or YMCAs, to fill the gaps in student and family needs. For example, Colorado School District 27J provides a webpage detailing “Monday Programming Opportunities and Special Discounts,” which includes expanded service by the Boys and Girls Club and other options, such as counseling for teens and physical activity programs.^32^ The Reeds Spring School District in Missouri cites a similar approach with the Boys and Girls Club, with the non‐school weekday serviced by a modified district‐run bus schedule to transport children to the program.^33^ Many programs are geared toward younger children, however, and many do not meet the particular needs of adolescents.

Another primary concern is the provision of food service to children on the non‐school weekday. Schools have multiple options in this regard. First, they could continue offering breakfast and lunch 5 days a week through the National School Lunch Program. However, this approach may negate any potential cost‐savings associated with reduced staffing on the off‐day, as staff would be needed to oversee food provision. This option also poses challenges to families who lack transportation options on non‐school days. Given that the 4 remaining school days are lengthened on a FDSW schedule, some schools have considered offering a third daily meal on school days. However, this may not mitigate any issues related to food security over the 3‐day weekend. Some community programs, such as the Boys and Girls Club, offer meals for participating children. Finally, schools could partner with organizations such as food banks or backpack programs to send students home with food for 3 days rather than the typical 2 days usually provided.^34^ However, this approach may have unintended consequences of stigma and shame,^35^ particularly for adolescents, and many food banks and other community organizations are strained by the ongoing COVID‐19 pandemic and may struggle to address this need for families and children.

Many of the issues that prompt districts to choose a FDSW would also complicate a response to the potential health implications of this school schedule. Specifically, budgetary concerns and transportation logistics for rural communities would also challenge the community to bolster support for families impacted by this school schedule. These challenges highlight the potential of the Whole School, Whole Community, Whole Child (WSCC) model.^36^, ^37^ The WSCC model emphasizes the role of the community in supporting school districts and encourages implementing evidence‐based strategies to promote health behaviors, such as linking schools with out‐of‐school programs, establishing joint‐use agreements for shared spaces like community kitchens and school gardens, and engaging community organizations to provide enrichment activities, all strategies that may help compensate for detrimental outcomes associated with the switch to a FDSW schedule. Nevertheless, without strong planning, the health needs of children attending FDSW schools may remain unmet, particularly in underserved communities.

### Human Subjects Approval Statement

This study was reviewed and approved by the Oregon State University Institutional Review Board (Study #8919).

### Conflict of Interest

The authors declare no conflict of interest.
